# Preclinical Imaging Evaluation of miRNAs’ Delivery and Effects in Breast Cancer Mouse Models: A Systematic Review

**DOI:** 10.3390/cancers13236020

**Published:** 2021-11-30

**Authors:** Francesca Maria Orlandella, Luigi Auletta, Adelaide Greco, Antonella Zannetti, Giuliana Salvatore

**Affiliations:** 1 IRCCS SDN, 80143 Naples, Italy; giuliana.salvatore@uniparthenope.it; 2 Institute of Biostructures and Bioimaging, National Research Council, IBB-CNR, 80145 Naples, Italy; luigi.auletta@yahoo.it (L.A.); antonella.zannetti@ibb.cnr.it (A.Z.); 3 InterDepartmental Center of Veterinary Radiology, University of Naples Federico II, 80131 Naples, Italy; 4 Department of Motor Sciences and Wellness, University of Naples Parthenope, 80133 Naples, Italy; 5 CEINGE-Biotecnologie Avanzate S.C.A.R.L., 80145 Naples, Italy

**Keywords:** breast cancer, miRNAs, mice models, preclinical imaging

## Abstract

**Simple Summary:**

The purpose of this systematic review was to assess the advancements in preclinical molecular imaging protocols used to study the delivery, tracking and therapeutic efficacy of miRNAs in mouse models of breast cancer. For this aim we have interrogated several browsers (PubMed, EMBASE, BIOSIS™ and Scopus) using the following terms: breast cancer, mouse, mice, microRNA(s) and miRNA(s). From 114 articles selected according to a PRISMA protocol, we focused on mouse models, routes of miRNA administration, therapy efficacy and molecular imaging. Importantly, we highlight here the advancements made in all imaging techniques’ applications used, providing a useful tool, on the basis of the current evidence, with which to suggest the best preclinical imaging protocol.

**Abstract:**

Background: We have conducted a systematic review focusing on the advancements in preclinical molecular imaging to study the delivery and therapeutic efficacy of miRNAs in mouse models of breast cancer. Methods: A systematic review of English articles published in peer-reviewed journals using PubMed, EMBASE, BIOSIS™ and Scopus was performed. Search terms included breast cancer, mouse, mice, microRNA(s) and miRNA(s). Results: From a total of 2073 records, our final data extraction was from 114 manuscripts. The most frequently used murine genetic background was Balb/C (46.7%). The most frequently used model was the IV metastatic model (46.8%), which was obtained via intravenous injection (68.9%) in the tail vein. Bioluminescence was the most used frequently used tool (64%), and was used as a surrogate for tumor growth for efficacy treatment or for the evaluation of tumorigenicity in miRNA-transfected cells (29.9%); for tracking, evaluation of engraftment and for response to therapy in metastatic models (50.6%). Conclusions: This review provides a systematic and focused analysis of all the information available and related to the imaging protocols with which to test miRNA therapy in an in vivo mice model of breast cancer, and has the purpose of providing an important tool to suggest the best preclinical imaging protocol based on available evidence.

## 1. Introduction

As has been recently estimated, breast cancer (BC) alone accounts for ~30% of all new diagnoses in women [1]. Although improvements in BC’s early diagnostic strategies and therapy have increased survival rates, this malignant tumor remains one of the most frequent causes of cancer-related mortality among females worldwide [1]. To date, it is well-known that BC is a complex and heterogeneous disease that can be classified into several subtypes based on histological and genetic characteristics. Through the combinations of molecular markers’ expression in the cancer cells, such as estrogen receptor (ER), progesterone receptor (PR) and human epidermal growth factor receptor 2 (HER2), it is possible to define principal intrinsic BC subtypes: luminal A, luminal B, HER2-enriched, basal-like/triple-negative and normal-like, which are characterized by different pathophysiology, prognosis and sensitivity to treatments [2,3].

Recent studies have reported that these different BC molecular subtypes are also associated with alterations in microRNAs’ expression and function [4,5,6]. MicroRNAs (miRNAs) are small non-coding molecules (18–22 nucleotides) that act on gene expression at the post-transcriptional level, contributing to the regulation of several biological functions. Indeed, through targeting the sequences in the 3’ untranslated region (UTR) of specific target mRNAs, miRNAs can induce the inhibition of translation or the degradation of their targets [7]. Consequently, based on target mRNAs’ activity, miRNAs have been defined as tumor suppressors or oncogenes (oncomiRs) [8]. Several studies have highlighted the prognostic and therapeutic roles of specific miRNAs in BC cells, and have also suggested their important role in the modulation of drug response or resistance [9].

In BC, miRNAs’ dysregulation has been demonstrated to promote malignant hallmarks such as proliferation, genome instability, cell invasion, drug resistance and metastasis. Thus, the restoration of these molecules’ expression using miRNA mimic or inhibitory sequences could become an essential point for the future development of novel therapeutic tools [10].

However, major drawbacks in using miRNAs as a therapy are the presence of nucleases in body fluids, which prevents the existence of any intact RNA free in the extracellular space, their rapid blood clearance, immunotoxicity and low tissue diffusion [11,12]. Indeed, it has been proven that miRNAs exist both intracellularly extracellularly; when they are secreted extracellularly, they are included in small vesicles called exosomes [11]. Thus, it is clear that miRNAs cannot be directly injected into the organism to be treated, hence demonstrating the need for the development of miRNA delivering systems. Some of the most frequently used systems for delivering miRNAs into target cells include inorganic nanomaterials (such as nanoparticles, NPs), lipid-based delivery systems or viral vectors [12,13]. The availability of these novel local and systemic delivery systems has allowed miRNAs to be exploited in clinical trials by restoring the expression of tumor suppressor miRNAs or by inhibiting the activity of oncomiRs [9].

Among the miRNA-based therapeutic strategies being tested in ongoing phase I trials, there is a specific mimic for the tumor suppressor miR-16 (MesomiR-1) for the treatment of mesothelioma [14]; the same is true for anti-miR-155 (MRG-106 Cobomarsen) in current phase I and II clinical trials for the treatment of lymphoma and leukemia [15]. The first clinical trial with MRX34, a mimic of miR-34 encapsulated in liposomal NPs, was discontinued due to severe adverse events. Thus, still to date, major obstacles to fully translating miRNAs in clinic are effective delivery and off-target effects.

Parallel to the therapeutic miRNA development procedure, advances in preclinical imaging, using mouse models, for evaluating miRNAs’ delivery have occurred in recent years.

Mouse models still represent an essential step in translating results from cell biology to the target species. The use of different preclinical molecular imaging techniques, in particular optical imaging, has significantly contributed to the investigation of the crucial role of miRNAs in BC progression and in evaluating miRNAs’ delivery to tumors, as well as their therapeutic effects. Molecular imaging (MI) allows for the non-invasive studying of the main cancer pathways in vivo, in real-time and in a quantitative way [16]. Using MI, it is possible to continuously obtain extensive information via the same animal, i.e., each animal acting as its own control, thus reducing the biological variability, number of animals required and cost for a particular study. The multimodality imaging approach provides anatomical and physiological complementary data that can improve the development of new anticancer drugs and more easily translate preclinical evidence into clinics [17]. Many studies have been performed using bioluminescence or fluorescence imaging integrated, in some cases, with morphological CT or MRI to assess the functions and effects of specific miRNAs [16].

The most innovative strategy in this field is the use of theranostic NPs that by integrating targeting, imaging and therapeutic abilities into one single nano-formulation allow drug accumulation to be monitored in real time to formulate disease diagnosis and evaluate treatment efficiency [18]. These multifunctional nanotheranostic platforms permit the visualization of tumor-specific miRNAs targeting and the evaluation of their effects on tumor growth and metastases formation.

Within this frame, despite the growing interest in and promising findings related to the potential of miRNAs in public health, still to date in the literature, to our knowledge, there is not an updated overview. Indeed, a review concerning recent advances in miRNAs as diagnostic and therapeutic agents using molecular imaging in preclinical mouse models of BC could help researchers to gain comprehensive knowledge regarding this topic.

Thus, in this systematic review, we explore the advancements by updating previous reviews and by applying preclinical molecular imaging to study miRNAs in mouse models of BC. Due to the versatility of MI, we looked for all possible imaging techniques’ applications, i.e., tracking the delivery and studying the efficacy of miRNAs as potential anticancer agents.

## 2. Materials and Methods

### 2.1. Literature Search Strategy

This systematic review was prepared according to both PRISMA (Preferred Reporting Items for Systematic Reviews and Meta-Analyses) and SYRCLE (Systematic Review Protocol for Animal Intervention Studies) guidelines and checklists [19,20].

Studies were searched on PubMed^®^ (including MEDLINE^®^), EMBASE, BIOSIS™ and Scopus using the following keywords: “Breast cancer” (and) “microRNA” (and) “mouse”.

**Figure 1:**
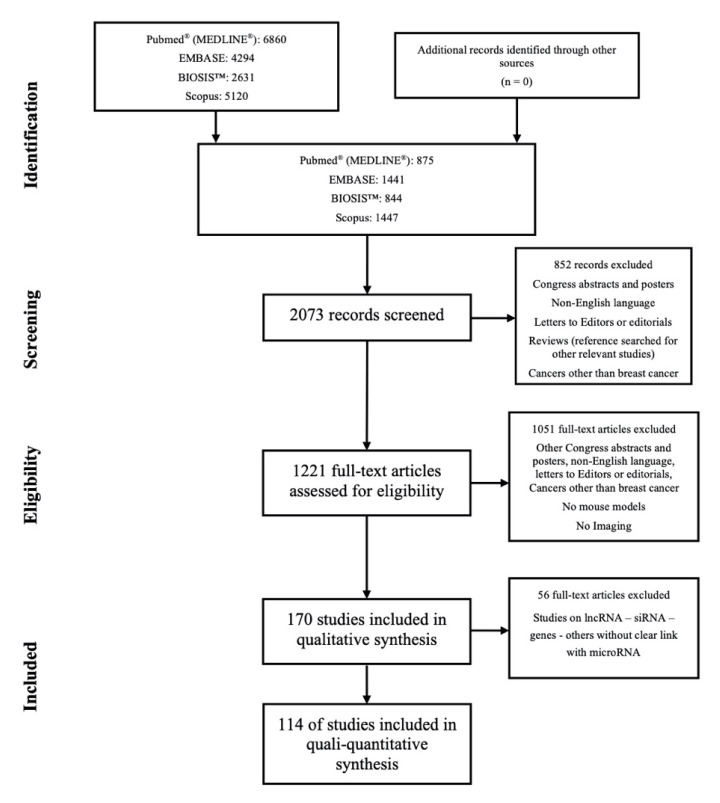
Flowchart for the strategy searches.

A total of eight search strings were applied in each database; indeed, searches were repeated using both singular and plural and using both “miRNA” and “microRNA”, “mouse” and “mice”. A PRISMA flow diagram [21] is reported in Figure 1.

All of the studies published in the last six years (2015–2021) which reported preclinical molecular and diagnostic imaging results in vivo or ex vivo in murine models of BC were included in this systematic review. Reference lists from relevant reviews identified in the database searches were manually searched to identify other eventual studies. Searches were concluded on 1 May 2021.

### 2.2. Study Selection and Eligibility Criteria

An electronic spreadsheet was prepared to report, for each study, the title, list of authors list, date of publication and language; moreover, whenever indicated, it was reported if the study was a review, an extensive research paper, an abstract or a letter/editorial.

All non-English language papers were excluded for the authors’ and readers’ convenience. After removing all duplicates, congress abstracts and posters, letters to Editors or editorials, each author was asked to screen the studies based on their titles and abstracts. Exclusion criteria in the screening phase were (i) article’s title not referring to cancer, (ii) article’s title identifying cancers other than BC and (iii) article’s abstract not including miRNAs.

Eligibility assessment was performed by all authors independently by screening the full texts. Inclusion criteria were (i) the use of in vivo or ex vivo molecular preclinical or diagnostic imaging, (ii) mouse models of BC, both xenografts, orthotopic and metastatic, and (iii) apparent miRNA involvement in the molecular processes studied. Both murine and human BC cell lines were included. Studies were considered not eligible if (i) ex vivo imaging was not performed on whole organs, but on histological samples, e.g., immunohistochemistry, as well as fluorescent confocal microscopy on tissues, (ii) models were produced in species other than mice (Mus musculus), or they were other than BC, and (iii) the study was on molecules other than miRNAs, e.g., long non-coding RNAs, small interfering RNAs, etc., or their effect on miRNAs could not be identified by reading the study. Since all five authors worked independently, the majority dictated if an article should have been included or not. Whenever an author identified an interesting article that was excluded, a consensus for eventual inclusion was reached by discussion between all authors.

### 2.3. Data Extraction

A.G., L.A. and A.Z. independently extracted from the selected studies (i) the mouse strain, (ii) the cell line used and all its peculiar characteristics, i.e., all eventual genetic modification of the original cell line, (iii) the model generated with the cell line, i.e., orthotopic, subcutaneous xenograft or metastatic, (iv) the miRNAs studied and their administration route, (v) the imaging modality or the multimodal approach used and (vi) the outcome measure, i.e., tumor volume reduction or changes in pathophysiologic aspects. Two other authors (G.S. and F.M.O.) independently extracted from the selected studies (i) the cell line used and all its peculiar characteristics, i.e., all eventual genetic modification of the original cell line, (ii) the model generated with the cell line, i.e., orthotopic, subcutaneous xenograft or metastatic, and (iii) the miRNAs studied, their administration route and the presumed effect. L.A. and F.M.O. reviewed and summarized all the information retrieved and discussed with all authors whenever discrepancies were detected and a consensus was needed.

## 3. Results

### 3.1. Literature Search

As reported in Figure 1, the search strategy using the eight research strings identified 6860 scientific manuscripts on PubMed^®^ (MEDLINE^®^), 4294 on EMBASE, 2631 on BIOSIS™ and 5120 on Scopus. The eight lists were compared within each search engine, deleting all duplicates, hence the final number of manuscripts was 875 for PubMed^®^ (MEDLINE^®^), 1441 for EMBASE, 844 for BIOSIS™ and 1447 for Scopus. At this point the four lists were merged together, erasing duplicates again, with a definitive list of 2073 records. Such a list was individually screened by each author, relying on the title and abstract, excluding all non-English papers, congress abstracts and posters, letter to editors, clear reference to cancers other than BC or to other pathologies at all in addition to reviews. Reviews were, however, searched for other relevant references, but no other eligible papers were detected. In this phase 852 records were excluded and 1221 papers were assessed for full text eligibility, excluding all those in which there was no use of mouse models of BC, there was not in vivo imaging or ex vivo on whole organs, i.e., were excluded imaging techniques applied to histological samples. At this point, 170 papers were studied to prepare the qualitative–quantitative synthesis, further excluding all manuscripts in which the animal models were used to study long non-coding, small interfering or other RNAs, as well as genes or other signaling molecules, without a clear link to a miRNAs. The final data extraction was from a total of 114 manuscripts.

### 3.2. Mouse Models of Breast Cancer

**Figure 2:**
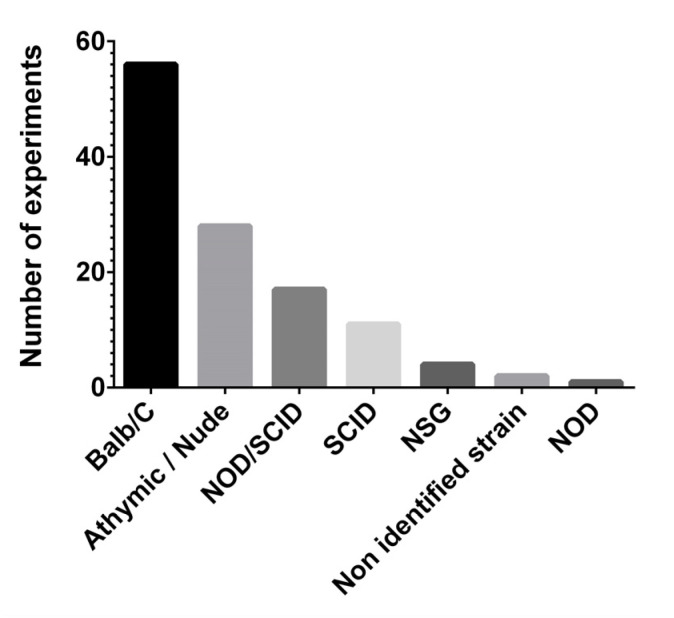
Number of experiments regarding the murine strain used in the articles analyzed. NOD: non-obese diabetic; SCID: severe combined immunodeficient mice; and NSG: NOD SCID gamma mouse.

Various factors play a role in the study of preclinical models, in particular the mouse strain, the cell line and the engraftment route. A summary of the murine strain used in the articles analyzed and the relative references are shown in Table 1, while Figure 2 shows the absolute number of experiments for each strain.

The murine genetic background most frequently used in miRNA studies has been determined to be Balb/C (46.7%), followed by different strains of athymic and/or nude mice (23.3%), non-obese diabetic, severely immunocompromised strains (NOD/SCID) (14.2%) and SCID strains (9.2%). Only a few experiments were performed on NOD/SCID gamma strains (NSG) (3.3%), and only one on NOD mice (0.8%). In two papers it was not possible to identify the murine strain used (1.7%) [124,125]. To be noted, only one was a transgenic model, the vascular endothelial growth factor receptor 2 (VEGFR2)-luc mouse. This model was generated, in the studied report, from an FVN/B strain, and it harbors the luciferase gene downstream from the VEGFR2 promoter region. In brief, anytime the VEGFR2 is transcriptionally activated, luciferase is transcribed as well; hence, this model allows the direct, non-invasive and quantitative monitoring of VEGFR2 via bioluminescence imaging (BLI) [126].

**Figure 3:**
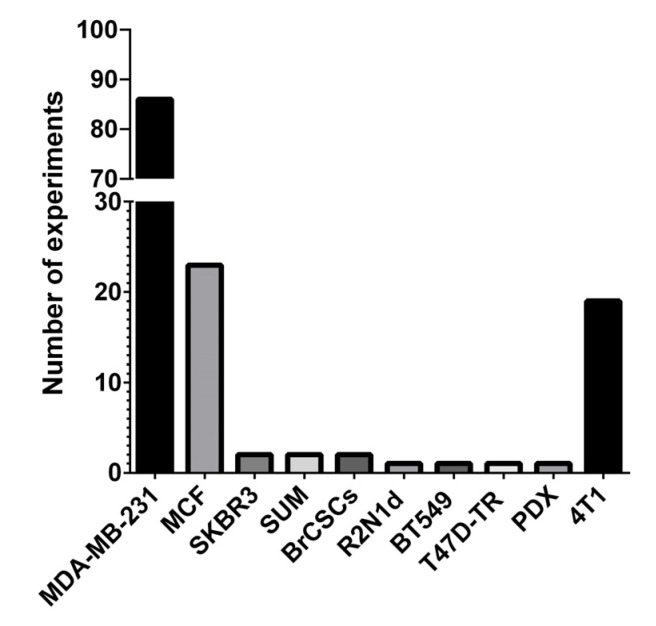
The absolute number of experiments regarding the cell lines, BrCSCs and PDX used to generate animal mice models. BrCSCs: breast cancer stem cells; PDX: patient-derived xenograft; and T47D-TR: tamoxifen-resistant.

Regarding cell lines, in most of the experiments human-derived cell lines were used, and only few used syngeneic, i.e., mouse-derived, cell lines of BC. Figure 3 shows the absolute number of experiments for each cell line, and Table 2 shows the different specific modification to each cell line and the relative references.

In detail, MDA-MB-231 was used in most experiments (62.5%), followed by MCF- (16.7%). SKBR3 and SUM-derived cells were used in two experiments each (1.4% each), whereas R2N1d—labeled with green fluorescent protein (GFP) and transfected with miR—, BT549—transfected with miR—and T47D-TR (tamoxifen-resistant) cell lines were used in one experiment each (0.7% each). Two experiments (1.4%) used breast cancer stem cells (BrCSCs): in one experiment, BrCSCs were obtained after the induction of the differentiation of BC cells purified from fresh tissues from patients’ mastectomies and then transduced with GFP via lentivirus infection, prior to being used in an orthotopic model [84]. In the second experiment BrCSCs were obtained from both patients’ tissues and from MDA-MB-231 and MCF-7 cell lines [59]. One experiment (0.7%) used a patient-derived xenograft (PDX) labeled with luciferin and modulated for miR precursor expression [95]. The only syngeneic cell line used was 4T1 (13.8%).

Regarding the murine model, the three models for cellular engrafting, i.e., subcutaneous and orthotopic xenografts as well as a metastatic model obtained by the intravenous injection (IV)of cancer cells, were all represented (Table 3).

The most frequently used model was the IV metastatic model (46.8%), which was obtained either via intravenous injection (68.9%)—in one paper it was indicated as intraarterial [58]—in the tail vein or in the left ventricle (9.8%). Direct intratibial injection to study osseous metastasis was applied in a few experiments (6.6%), as was direct intrapulmonary injection (1.6%). Finally, the development of spontaneous metastasis after orthotopic injection was obtained either after surgical resection of the primary nodule (4.9%)—with lymph node [112] or pulmonary metastasization [23,34]—or with the primary orthotopic implant on site (8.2%). In two papers, it was not specified how the metastatic model was obtained [70,106].

The orthotopic model, with injection in the second or forth mammary gland or fat pad, transcutaneously, after surgical exposure or intra nipple, was the second most frequently used model (29.2%). A subcutaneous xenograft, implanted in various sites, i.e., on the shoulder, the armpit, the flank and thigh, was used in 24% of the experiments.

When interpreting tables and results, it should be noted that various reports performed multiple experiments using different cell lines and/or multiple models and/or multiple mouse strains, for all of which imaging was applied [23,25,34,43,48,50,51,53,54,56,57,74,76,78,81,85,86,89,95,98,102,108,120,128,131].

### 3.3. Mode and Route of Therapy Administration

**Figure 4:**
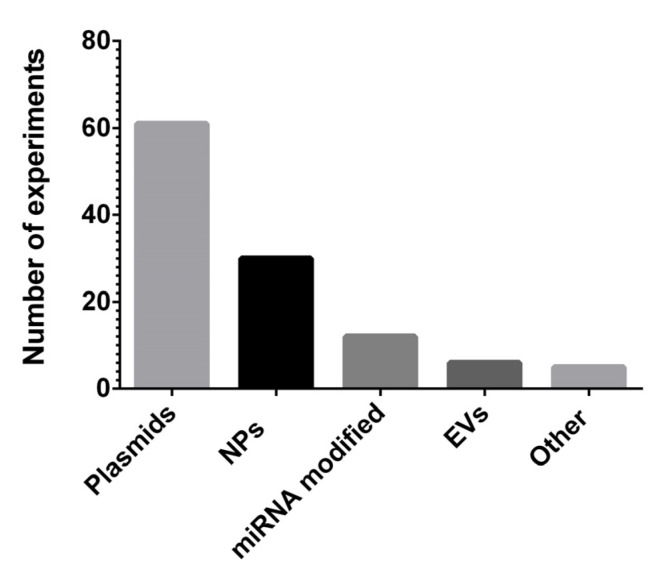
The absolute number of experiments conducted for each miRNA delivery system. NPs: nanoparticles; EVs: extracellular vesicles.

In this paragraph we systematically report the different in vivo modalities and routes of miRNA therapy administration. In Figure 4 the absolute number of experiments done for each delivery system is shown, while in Table 4 the miRNAs used, the specific formulations of vehicle system and the relative references are reported.

Most of the preclinical mice models (54.4%) were generated by injecting luciferase (Luc)-labeled BC cells transfected with DNA or lentiviral plasmids. In detail, a lentiviral vector was used to modulate the expression of miR-206 [95], -1 [70], -124 [131], -211-5p [128], -494 [78], -1204 [53], -133b [48,54], -101 [25], -630 [90], -150 [104], -133a-3p [49], -452 [62], -543 [80], -96 [22], -29a [55], -455-3p [77], -30a [127], -100 [84], -548j [86], -940 [71], -429 [51], -442a [88], -373 [89], -509 [121], -190 [79], -125b [42,106], -125a-5p [72,73], -33a [120], -33b [23], -138 [100], -27b [134], -454-3p [57], -23a [87] and -218-5p [130], as well as of miR-30 family members (miR-30a-b-c-d-e) [58]. A lentiviral vector was also generated to express a circular inhibitor miRNA (CimiRs) specific to silencing the expression of miR-223 and miR-21 [118].

Moreover, BC cell lines were transfected with DNA constructs encoding for the following miRNA precursors and/or inhibitors: let-7a-5p [31], miR-196a [44], -205 [114], -361-5p [56], -590-3p [119], -567 [117], -106b-5p [52], -497 [126], -135/-203 [76], -29/-30 [30], 14q32-encoded miRNAs [83] and miR-191/425 cluster [43]. The effect of miR-1 overexpression was studied both in mice injected with MDA-MB-231-luc cells stably transfected with miR-1 precursor and in tumor-bearing mice treated with the synthetic miR-1 mimic [91].

In eight studies BC cells were transfected with different types of plasmid (lentiviral or DNA) encoding mimics and/or inhibitors specific for miR-200 family members (miR-200a, -200b, -200c, -141 and -429) [50,81,85,102,103,110,116,129].

In one experiment a doxycycline-inducible vector was used to overexpress miR-301a-3p [75].

Nanoparticle (NP)-based delivery represents a promising strategy for BC treatment, preventing miRNA degradation in the bloodstream and improving miRNA delivery in tissue-specific targeting. Indeed, we found that 29 experiments (25.4%) were conducted using different formulation of NPs, including natural lipid-based NPs (LNPs) and synthetic NPs composed of inorganic materials such as silica (SiO_2_), gold (Au) or polymer (e,i. polyamidoamine—PAMAM—dendrimers) [137] (Table 4).

Organic LNPs were generated to encapsulate miR-203 mimic [60], AgomiR-143 [36], AgomiR-186-3p [37] and the “edited” form of miR-379-5p [123]. AntagomiR-214-3p was loaded into the osteoclast-targeting delivery system (D-Asp8-liposome) [133].

Inorganic synthetic NPs was engineered to encapsulate miR-145 using PAMAM dendrimers modified with a thioaptamer (TA), a protein that binds CD44—receptors highly expressed on BC cells [38]. Poly(ethylene glycol)–polyethylenimine (mPEG–PEI) was complexed with a molecular beacon (MB) to detect miR-34a in BC [125].

Gold nanoparticles (AuNPs) were used to deliver miR-708 [34] and miR-96/-182 [35] mimics; other AuNPs were formulated with a photoacoustic (PA) nanoprobe that released a PA signal in the presence of the oncogenic miR-155 [27]. Magnetic (MN) NPs were engineered for the recognition of specific oncomiRs in BC tissue [39,111] or conjugated with locked nucleic acid (LNA) to inhibit the activity of miR-10b [26,112]. SuperparaMN iron oxide NPs (SPIONs) conjugated with Argonaute-2 protein (AGO2) were formulated to deliver miR-376B mimic in BC tissue [122].

In five independent studies, the activity of the tumor suppressor miR-34a was replenished using: (i) hTERT promoter-driven VISA liposomal NPs [59]; (ii) polymeric hybrid nanomicelles simultaneously delivering doxorubicin (Dox) [28]; (iii) dextrin-PEI-CM nanoplex (DPC) also delivering a cyclam monomer (a CXCR antagonist) [65]; (iv) silica dioxide NPs (SiO_2_NPs) [99]; and a (v) lipid core–shell nanocarrier coated with cationic albumin co-delivering docetaxel [135].

In five studies, miR-21 inhibition was obtained in vivo using: (i) a core of phi29 pRNA- three-way junction motif (3WJ) harboring the RNA aptamer for EGFR (3WJ/EGFRapt/anti-miR21) [132], (ii) a core of 3WJ harboring the aptamer binding to CD133 receptor (3WJ/CD133apt/anti-miR21) [113], (iii) polydopamine (PDA)-based NPs [68], (iv) tumor extracellular vesicles complexed with gold-iron oxide NPs (TEV-GIONs) [107] and (v) RNA nanospheres into nanopompons [47].

In a few studies, multiple miRNAs were simultaneously co-delivered using polymeric NPs triggered in BC tissue by the urokinase plasminogen activator peptide (uPA) [108], by ultrasound [33] or by RNA-triple-helix hydrogel scaffolds [82].

The combined delivery of miRNA and a chemotherapeutic drug into tumor sites was obtained using polymeric hybrid NPs (Dox + miR-34a) [28], polydopamine (PDA)-based NPs (Dox + antisense-miR-21) [68], magnetic NPs (Dox + miR-10b) [112], calcium/phosphate lipid NPs (paclitaxel + miR-124) [66] and a lipid nanocarrier coated by cationic albumin (docetaxel + miRNA-34a) [135]. Interestingly, specific NPs were developed to co-deliver the photosensitizer indocyanine green (ICG) and the inhibitor of miR-21 [41].

In 12 studies (10.5%) we found that to enhance the systemic delivery efficacy of mimic/inhibitor miRNAs, in the absence of a protective vehicle, synthetic small molecules or chemical modifications are added to miRNAs, increasing their stability in the circulatory system. “CMM489” is a chemically modified mimic in which uracil in the guide strand of the miR-489 tumor suppressor was reply with 5-fluorouracil (5-FU) [109]. A single-strand miRNA inhibitor (“AntagomiR”) and a double-stranded mimic (“AgomiR”) are RNAs harboring bases that are chemically modified to overcome the RNA instability. In this context, mice were treated with AgomiR-338-3p [29], AntagomiR-16-1-3p [32] or with AntagomiR-100 [24]. Additionally, FolamiR-34a is a modified mimic in which a folate group was attached to a miR-34a sequence to directly bind the BC cells overexpressing the folate receptor [115]. Another example of artificially synthesized nucleic acid is represented by the peptide nucleic acid (PNA) labeled with [^99m^Tc] that recognizes, in vivo, the presence of oncomiR-155 [45]. Finally, the inhibition of the activity of miR-21 [40,63,94], miR-210 (“Targapremir-210”) [96], miR-544 [97] and miR-10b (“Linifanib”) [105] was obtained using small-molecule compounds.

Exosomes are small extracellular vesicles (EVs) of 30–150 nm in diameter, which are released by cancer cells in the tumor microenvironment for intercellular communication. In six studies (5.3%), researchers have exploited the possibility to use exosomes to encapsulate the following miRNAs: let-7 [61], miR-210 [69], -335 [101], -159 [46], -4443 [74] and anti-miR-21 [107].

Recently, the anticancer activity of miRNAs derived from marine invertebrate *marsupenaeus japonicus* shrimp was analyzed in two experiments in which tumor-bearing mice were fed shrimp that were fed mja-miR-35-expressing bacteria [64] or treated with synthesized shrimp miR-34 [93].

### 3.4. Therapy Effect and Efficacy

**Figure 5:**
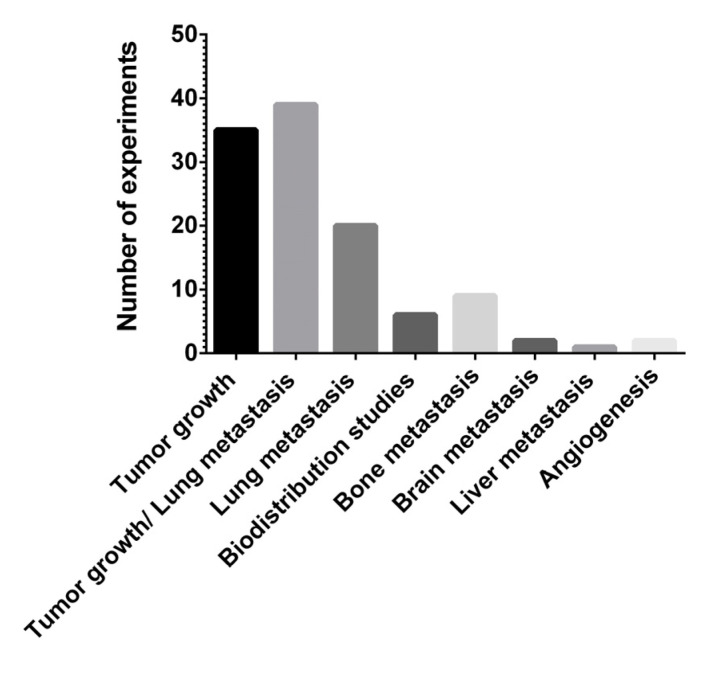
The absolute number of experiments regarding the effects of therapy and its efficacy are shown.

The potential role of miRNAs could be categorized based on their mode of action and therapeutic efficacy established in preclinical BC mouse models. The number of experiments and references regarding the effects of therapy and its efficacy are summarized in Figure 5 and in Table 5.

Among the biological effects reported in mice, tumor growth alone (30.7%) or in combination with tumor metastasis (34.2%) are determined to be the effects most frequently studied.

Indeed, tumor growth inhibition occurred in tumor-bearing mice intravenously injected with several miRNAs (let-7, miR-145, -335, -34a, -203, -376B, -205/anti-miR-221, -379-5p and anti-miR-21) delivered using different approaches, such as NPs [33,38,60,68,82,99,111,113,122,123,132] and extracellular vehicles [46,61,101,107]. The inhibition in tumor growth occurred in mice injected with BC cells transfected with miR-442a [88], -100 [84], -27b [134], -567 [117], -455-3p [77], -301a-3p [75], AntagomiR-138 [100] and cirBulg21/223 [118] compared to mice injected with BC cells transfected with a control plasmid. On the contrary, miR-196a overexpression in MDA-MB-231-luc cells promoted this capability [44].

Tumor growth was impaired in tumor-bearing mice treated with linifanib [105], TargapremiR-210 [96], small-molecule “1” (specific for miR-544) [97], FolamiR-34a [115], trichostatin A (an inhibitor of histone deacetylase that up-regulates miR-125a-5p) [72], “CMM489” (a chemically modified miR-489) [109] or shrimp miR-34 [93].

Interestingly, the injection of lipid vehicles loaded with AgomiR-186-3p [37] and AgomiR-143 [36] inhibited tumor growth and reduced the uptake of [ 18F]-fluoro-deoxyglucose ([18F]-FDG).

Regarding the effects of miRNA delivery on either tumor growth or lung metastasis, we found that luciferase expressing BC cells transfected with miR-101 [25], -141 [116], -361-5p [56], -30a-5p [127], -125a-5p [73], -1 [91], -211-5p [128], -190 [79], -206 [95], -33b [23], -33a [120], -96 [22], -133b [54], -1 [70], -494 [78], -29b/-30d [30], anti-miR-1204 [53] and miR-191/-425 sponge [43] exerted antitumor and metastatic activity compared to BC cells transfected with an empty vector. The silencing of let-7a-5p [31] and of miR-16-1-3p [32] in MDA-MB-231 and of miR-338-3p [29] in 4T1 cells influenced tumorigenesis and lung metastasis after implantation in nude mice.

The effects of miR-122 on glucose metabolism, tumor growth and metastasis were evaluated in different animal models using luciferase-labeled BC-transfected cells or EVs containing miR-122 [98]. Antitumor and antimetastatic effects were evaluated after the injection of NPs loaded with specific miRNAs (-34a [65], -96/-182 [35], -708 [34], antimiR-21/-10b [108] and AntagomiR-10b [26]) or following treatment with AC1MMYR2 (a specific small-molecule inhibitor of miR-21) [40], AntagomiR-100 [139] or with the antioxidant pterostilbene [92]. A novel approach was reported by Wu and colleagues, in which the co-delivery of miR-21 inhibitor and indocyanine green (ICG) exerted anticancer activity, photokilling MDA-MB-231 cells [41].

Twenty animal models (17.5%) were conducted to study the effects of miRNA delivery on lung metastasis, and only a few experiments were performed analyzing bone (7.9%), brain (1.7%) and liver metastasis (0.87%).

Lung metastasis was suppressed when BC-luc cells were transfected with the following miRNAs: miR-630 [90], -452 [62], -590-3p [119], -150 [104], -543 [80], -133a-3p [49], -133b [48] and 14q32 microRNA cluster [83], or transfected with the inhibitors for miR-106b-5p [52], -23a [87] and -454-3p [57], or when mice were injected with shrimp miR-35 [64], with a small molecule that binds the precursor of miR-21, activating its destruction [94]. On the contrary, an increased incidence of metastasis was established in mice injected with BC cells overexpressing miR-29a [55] and -373 [89]. miR-548j overexpression increased the metastatic potential of BC cells without affecting tumor growth [86]. Five studies reported that miR-200 family members (miR-200a, miR-200b, miR-200c, miR-429 and miR-141) play an important role in the formation of the primary tumor and in the metastatic phenotype of BC [50,85,102,110,129].

The co-delivery of miRNA and small-molecule chemotherapy drugs in tumor sites represents a promising strategy to fight the progression of cancer in mice. In this context, the co-delivery of Dox with miR-34a [28] or with miR-159 [46] to cancer sites suppressed tumor growth. A regression of lung metastasis disease was established by the cotreatment of miR-10b and Dox [112]. The combination treatment of taxol and AC1MMYR2 (a small molecule that reduces miR-21 expression) [63] or of miRNA-34a and docetaxel [135] impaired tumor growth and metastasis. Paclitaxel and miR-124 coloaded in a lipid nanosystem impaired lung metastasis formation in orthotopic mice [66]. Co-delivery of miR-96/-182 with cisplatin, using NPs, reduced primary tumor formation and prevented lung metastasis formation [35].

Two experiments reported that brain metastasis formation was affected, in vivo, by the modulation of miR-509 [121] and miR-141 [81]. In only one study was liver metastasis impaired by the administration of EVs carrying miR-4443 inhibitor [74]. Bone metastasis was impaired by the overexpression in BC cells of miR-124 [131], -429 [51], -205 [114], -940 [71], -125b [106] and -30 family members [58], the inhibition of miR-218-5p [130] or by intratumorally injecting synthetic miR-135/-203 mimics [76] or osteoclast-targeting AntagomiR-214-3p using (D-Asp)8-liposome [133].

Currently, the detection of miRNAs in cancer tissues could help to monitor the progression of cancer. From our research, biodistribution studies were found in six articles (5.3%). miR-155 expression was monitored in two different studies by intravenously injecting a PA nanoprobe [27] and by the synthesized peptide nucleic acid (PNA) mimic loaded with [^99m^Tc] [45]. A molecular beacon (MB) circuit was developed to monitor the expression of miR-34a in BC tissue with high sensitivity [125]. A nanosensor conjugated with MN-NPs allowed for the discrimination of BC cells from non-tumoral cells based on miR-10b expression [111]. Monitoring the expression of miR-200c [103] and of miR-14/-21/-9 [39] in tumor-bearing mice was useful to determine the therapeutic approach. Finally, tumor angiogenesis was evaluated in three studies reporting that miR-497 exhibited anti-angiogenesis and antitumor effects targeting VEGFR2 [126] and miR-210 promoted angiogenesis [69], while miR-125a-5p affected tumorigenesis, metastasis and angiogenesis in vivo [73].

### 3.5. Molecular Imaging

**Figure 6:**
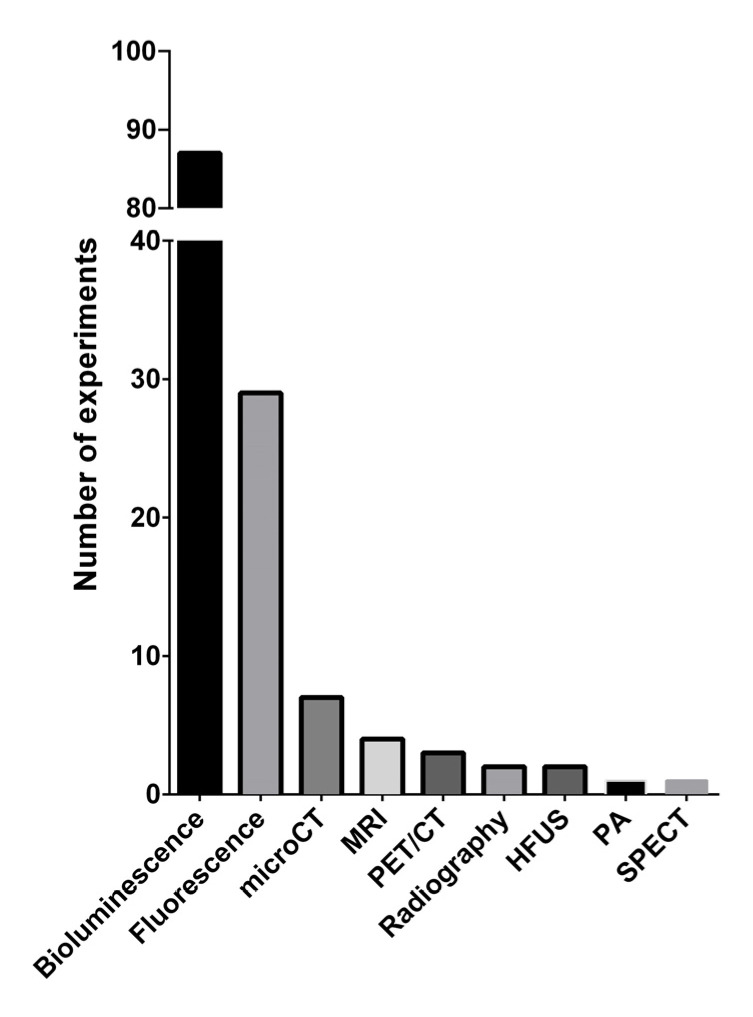
The absolute number of experiments for each imaging modality used to analyze the biodistribution and therapeutic effects of miRNAs’ delivery in mice. CT: computed tomography; MRI: magnetic resonance imaging; PET: positron emission tomography; HFUS: high-frequency ultrasonography; PA: photoacoustic; and SPECT: single-photon emission computed tomography.

Most of the known preclinical imaging techniques have been applied in studying miRNAs’ delivery and/or efficacy. Figure 6 shows the absolute number of experiments for each imaging modality, and Table 6 shows the number of experiments for each specific modality and aim, in addition to the relative references.

Bioluminescence was determined to be the most frequently used tool (64%); this technique was used as a surrogate for tumor growth for efficacy treatment or for the evaluation of tumorigenicity in miRNA-transfected cells (29.9%); for tracking, evaluation of engraftment and response to therapy in metastatic models (50.6%); and for both the aforementioned aims in the same experiment, evaluating metastasis either in vivo or ex vivo on whole organs (16.1%). As already reported, in one experiment (1.1%) a transgenic VEGFR2-luc mouse was used to evaluate the expression of VEGFR by non-invasive bioluminescence, and to evaluate the effect of miRNA mimic treatment as anti-angiogenetic therapy [126]. Bioluminescence was also used for vector uptake and intercellular target repression (2.3%), although most of these experiments were performed by fluorescence imaging.

Fluorescence imaging was the second most frequently used technique (21.3%), and was used primarily to trace vector biodistribution (73.2%) by using different strategies, e.g., by directly conjugating the miR to the fluorophore, or simply uploading the fluorophore within the vector. In one interesting report, the vector was neither an NP nor an extracellular vesicle nor a liposome, but a folate, directly linked to the miR as well as to a near-infrared (NIR) fluorophore for fluorescent detection [115]. Fluorescence was rarely used for tumor growth evaluation (11.5%), analysis of tumor persistence after direct intratumoral injection of the miR labeled with fluorophore [41] or within fluorescent SiO_2_ NPs [99] (7.7%), and for cell tracking (3.8%). One interesting experiment (3.8%) showed the ability of a molecular beacon to detect and image endogenous miRNAs with a high level of specificity in vivo [125]. Besides these two mostly used imaging techniques, other tools were used to study the biodistribution or different aspects of miRNAs’ treatment efficacy. Microcomputed tomography (µCT) was used to analyze in vivo or ex vivo osteolytic lesions in metastatic bone models or identify pulmonary metastases (5.2%). The former evaluation was performed with standard radiography (1.5%) in two other experiments [58,131]. Magnetic resonance imaging (MRI) was used in 2.9% of the experiments, mainly for the detection of magnetic NPs’ biodistribution, and only in one experiment for the evaluation of invasiveness of adjacent tissue [74]. Positron emission tomography (PET)/CT was applied with [18F]-FDG administration to evaluate tumor growth, in terms of tumor glucose metabolism, or for the detection of pulmonary metastases (2.2%). High-frequency ultrasonography (HFUS) was performed to evaluate tumor growth or microbubble-mediated nanoparticles delivery as a therapeutic intervention (1.5%). Photoacoustic (PA) (0.7%) imaging was used to determinate the ability of self-assembling nanoprobes to identify specific miRNAs. In brief, in the presence of a specific miRNA, aurum aggregation from the nanoprobes, via a hybridization chain reaction, allowed for the identification of the PA signal [27]. Finally, single-photon emission computed tomography (SPECT) (0.7%) was used to label and track a molecular probe and to evaluate both the specificity in detecting the selected miR and both for biodistribution purposes [45].

In addition to what has been already stated, a multimodal imaging approach, i.e., the use of multiple imaging technologies to evaluate different aspects or models within the same manuscript, was used and evaluated in 19 papers (16.7%) [26,28,33,34,35,36,51,58,69,74,83,106,107,111,112,130,131,132,133]. Finally, it is important to highlight that in some manuscripts in which multiple animal model are developed, the growth of the primary tumor was evaluated exclusively by tumor caliper measurement or tumor weighting ex vivo, whereas imaging (in vivo or ex vivo) was applied only for metastasis evaluation [22,29,30,31,32,42,43,53,54,56,58,70,78,91,93,102,116,127,128]. In other studies, the therapeutic effects of miRNA delivery were evaluated independently from their biodistribution visualization obtained with preclinical imaging [28,47,60,61,65,66,68,99,113,115,122,135].

## 4. Discussion

We systematically analyzed the most recent studies using preclinical imaging technologies to investigate the potential of specific miRNAs as therapeutic and diagnostic tools in BC. Although several systematic reviews focused on the crucial role played by miRNA in BC biology and as therapeutics [140], to our knowledge this review is the first systematic review that specifically focused on the use of preclinical molecular imaging for the evaluation of miRNAs’ delivery and effects in BC. Numerous are the advantages offered by the application of different imaging techniques to study animal models of cancer [16]. First, the possibility to perform in real time non-invasive longitudinal studies of the same mice. This allows the number of animals to be analyzed to be reduced, in accordance with Directive 2010/63/EU, the principle of the 3Rs (Replacement, Reduction and Refinement), and animal welfare considerations. It is noteworthy that it is the translational aspect of preclinical imaging that may be considered as a bridge from basic to clinical research. In this context, this systematic review documented all investigations that used different imaging technologies dedicated to small animals, such as optical imaging (OI), HFUS, MRI, CT and PET/CT, to evaluate and validate miRNAs as anti-cancer agents as well as shed light on molecular mechanisms.

Most of the studies included in this systematic review were performed on miR-10b (five studies), miR-21 (eleven studies), the miR-34 family (eight studies) and the miR-200 family (ten studies). A high level of miR-10b indicates a poor prognosis in BC, correlating with angiogenesis and metastatic behaviors (increased tumor size, lymph node positivity and a high Ki-67 score) [141,142,143]. Importantly, in NOD-SCID mice, a high miR-10b level led to distant metastasis, while in the 4T1 mouse mammary tumor metastasis model the delivery of AntagomiR specific for the silencing of miR-10b suppressed the distant metastasis [144,145]. Overexpression of miR-21, one of the most studied oncomiRs in BC, is associated with lymph node metastasis, resistance to anticancer agents and a poor prognosis [146,147,148]. The up-regulation of miR-21 in this cancer induces the silencing of several tumor suppressor genes, such as programmed cell death 4 (PDCD4) [149] and leucine zipper transcription factor-like 1 (LZTFL1) [150]. A tumorigenicity assay was recently performed in Balb/c-nude mice inoculated with BC cells silenced for miR-21 using specific peptide nucleic acids (PNA). In vivo, functional studies showed that PNA-antimiR-21 inhibits tumor growth in vivo [151]. Another important family correlated to cancer is the miR-34 family: it is comprised of miR-34a, miR-34b and miR-34c. They exert a tumor suppressor role in various cancers and are regulated by p53 [152]. In BC, miR-34a plays a crucial role in proliferation, motility and stemness [153]. Identified targets of miR-34a are SIRT1 and BCL2 [154]. Another example of oncomiR is miR-200a, which promotes epithelial–mesenchymal transition (EMT), drug resistance and metastasis by targeting tumor protein P53-inducible nuclear protein 1 (TP53INP1) and yes-associated protein 1 (YAP1) in human BC [155,156]. Importantly, miR-200a belongs to the miR-200 family that appears to be crucial for BC progression. In particular, the miR-200 family is composed of five members (miR-200a, miR-200b, miR-200c, miR-141 and miR-429) that are reported to be involved in EMT and angiogenesis of BC cells [157,158,159]. Besides, it is reported that deregulated levels of miR-200a and miR-200c occurred in the tamoxifen-resistant BC model, where they induced a reduction in the mRNA of c-MYB [159].

A major problem in the clinical use of miRNAs is the delivery method. This is due to several reasons, such as the destabilization of the RNA in circulation due to serum ribonucleases, ineffective targeting to the tumor cells because of the tumor microenvironment and a poor uptake of the miRNA. Several delivery methods have been tested, such as the lentiviral- and liposomal-mediated delivery of the tumor-suppressive miRNA miRNA-34a (miR-34a), which reduces the tumor burden in non-small-cell lung cancer (NSCLC) mouse models [160]. In addition to vehicle- and viral-mediated miRNA delivery, systemic injection of vehicle-free oligonucleotides has also been tested. However, this approach has proven problematic because of the pharmacokinetic and stability limitations associated with intravenous delivery, and thus either relies on local delivery or necessitates achieving a high oligonucleotide concentration that is often only seen in the kidneys and liver. Although local delivery is an option, achieving delivery beyond sites that are accessible to local delivery, such as to micrometastatic lesions, is not achievable.

Most of the imaging studies reported in this review used optical imaging (OI) to analyze miRNAs’ effects on cancer murine models, in particular bioluminescence (BLI) (64%) and fluorescence (21.3%). One of the most common applications of OI is to monitor tumor growth and metastasis formation in orthotopic xenograft models and transgenic animal models [161]. Furthermore, it is a highly sensitive technique and allows non-invasive monitoring of disease-relevant processes and permits the tracking of cells [162]. The main advantages of OI compared to other imaging platforms are the low cost and the absence of ionizing radiation, as well as the possibility to more easily translate the observations obtained in vitro on the corresponding cell line injected into animals. Fluorescence shows some disadvantages due to background signals and autofluorescence, which are absent in the BLI, which in turn has brightness and low spatial–temporal resolution [161]. Among the numerous studies analyzed in this review regarding BLI with luciferase to monitor tumor growth and/or metastatic spread, it is worthy to note the investigation that used VEGFR2-luc transgenic mice to monitor the effect of miR-497 mimic not only on tumor growth but also on tumor angiogenesis [126]. The results demonstrated that overexpression of miR-497 showed inhibitory effects on VEGFR2 activation [126]. The limits of fluorescence can be overcome by using near-infrared fluorophores that penetrate deeper into tissues and exhibit very low autofluorescence. An interesting study conjugated microRNAs to folate (FolamiR) for delivering them into cells that overexpress the folate receptor. In particular, the tumor-suppressive FolamiR, FolamiR-34a, was labeled with NIR fluorophore, and its delivery to TNBC xenografts was evaluated by OI [115]. Furthermore, Tu et al. reported a novel strategy for miRNA detection through enzyme-free signal amplification by self-circulation of the hybridization between the miRNAs and molecular beacon (MB) circuits. This approach allowed miRNA to be detected in the BC xenografts by amplifying the fluorescence signal and contributing improving detection sensitivity [125].

HFUS is the most suitable technique to monitor tumor growth due to the capability of this technique to perform accurate morphologic imaging. Most interesting, HFUS has also been used to deliver directly therapeutic microRNAs (AmiR-21 and miR-100) and TK-p53-NTR triple therapeutic gene, co-loaded in PLGA-PEG-PEI polymer NPs to tumor models of TNBC [33]. As our research group has demonstrated in past experiments, ultrasound mediated therapy, enhanced vascular permeability and microbubbles cavitation improve drug delivery directly into tumor sites [163]. PET has also been used to evaluate the response to miRNA therapy in a tumor model of TNBC by targeting tumor glycolysis [36], as well as to assess metastasis in an in vivo mice model of TNBC. The limitation of this technique is related to the high-cost relative to the radiotracer and/or to the necessity of having a cyclotron close to the animal facility, plus the necessity of using radiations. Thus, specific and long training is necessary to have a person able to perform experiments of nuclear medicine. An emergent methodology is high-field MRI, which combines the possibility of performing a morphologic analysis of the primary tumor to follow the spread of metastasis, plus the possibility of therapeutic delivery of miRNAs or miRNAs combined with chemotherapeutic agents with magnetic NPs. MRI has the advantage of avoiding the use of ionizing radiation, but has the disadvantage of requiring a long time for the acquisition of high-quality diagnostic images. A good anesthetic protocol and continuous monitoring of the mice model could compensate for the last issue. Finally, what mainly emerges from the cited papers, is the advantage that we could obtain by having a multimodal imaging approach to diagnose both the mice model of BC and to perform an efficient therapy. The last issue could also be better addressed through a targeted nanosystem with directed delivery against molecular markers of breast cancer.

## 5. Conclusions

The studies reported and discussed in this systemic review highlight the utility of preclinical molecular imaging focused on the development of novel therapeutic strategies for miRNAs based in breast cancer management. To date, despite the multiple advances in imaging technology, this extensive and focused literature review shows that optical imaging remains the most widely used method in preclinical investigations, probably due to its low cost and ease of use. In fact, only few of the papers we cited demonstrated the advantages that we could obtain by having a multimodal imaging approach to diagnose mice models of BC and perform an efficient therapy. Therefore, given the large amount of information that can be extrapolated from multimodal imaging and its strong translational power to the clinic, future studies using multiple imaging modalities are desirable. Finally, the development of NPs engineered to encapsulate miRNAs alone or in combination with other drugs and their delivery to specific targets will provide deeper knowledge in this research field, and will be certainly be one of the fields that will be improved in the future.

We aimed to highlight the role of preclinical imaging and its potentiality to test new experimental therapies for breast cancer patients, aiding the translation from in vitro studies to the clinic. Preclinical imaging is a continuously evolving field, and new nanoprobes could represent novel systems for personalized therapy in the future.

## Figures and Tables

**Table 1:** Murine strains used in miRNA experiments.

Background	Strain	No. of Experiments	References
Balb/C	As it	12	[22,23,24,25,26,27,28,29,30,31,32,33]
	/J	1	[34]
	cAnNCr	1	[35]
	athymic nude	3	[36,37,38]
	Nude	31	[30,32,39,40,41,42,43,44,45,46,47,48,49,50,51,52,53,54,55,56,57,58,59,60,61,62,63,64,65,66,67]
	-nu	1	[68]
	-nu/nu	1	[69]
	-nu/nu athymic	1	[70]
	cAJcl-nu/nu	1	[71]
	cAnN.Cg-Foxn1nu/Crl-Narl	2	[72,73]
	cJNju-Foxn1nu/Nju	1	[74]
	nude athymic CAnN.Cg-Foxn1nu/Crl	1	[75]
SCID	As it	7	[56,76,77,78,79,80]
	Beige	2	[54,81]
	CB17.Cg-PrkdcscidHrhr/IcrCrl	2	[34,82]
NOD	-Prkdc^em26^I/2rg^em26^/Nju	1	[28]
NOD/SCID	As it	14	[76,83,84,85,86,87,88,89,90,91,92,93,94,95]
	NOD.CB17-Prdkc^scid^/J	1	[82]
	B6.CB17-Prkdc^scid^/Sz	2	[96,97]
NSG	NOD/SCID/IL2Rγ-null	1	[98]
	NOD.Cg-Prkdc^scid^Il2^rgtm1Wjl^/SzJ	2	[99,100]
	NOD *scid* gamma	1	[101]
Athymic/nude	Nude (Nu/Nu)	8	[102,103,104,105,106,107,108,109]
	Athymic nude	2	[110,111]
	Athymic nu/nu	1	[111]
	Nude (NIH III nude)	1	[112]
	NCrnu/nu	1	[113]
	Athymic NCrnu/nu	1	[114]
	Nu/Nu (NU-Foxn1nu)	1	[115]
	Athymic Nude-Foxn1nu nude (NCI)	1	[116]
	Athymic Nude-Foxn1 nu/nu	1	[117]
	Nude (mice not furtherly identified)	10	[23,31,43,53,78,118,119,120,121,122]
	J:NU (outbred athymic nude)	1	[123]

**Table 2:** Cell lines used in miRNA experiments.

Cell Line	Derived	Labeling	Transfection	No. of Experiments	References
MDA-MB-231	Parental			17	[26,28,30,36,41,45,46,47,60,61,65,66,74,87,93,97,127]
	Parental		miR	14	[23,31,32,48,50,51,53,56,71,80,91,104,128,129]
	Parental	GFP		1	[67]
	Parental	GFP-luciferase		3	[92,105,108]
	Parental	GFP-luciferase	miR	3	[49,83,110]
	Parental	GFP	miR	1	[102]
	Parental	Luciferase	miR/antimiR	17	[31,32,44,54,62,76,78,79,86,100,103,117,119,120,127,130,131]
	Parental	Luciferase		15	[30,40,43,63,64,82,91,93,94,96,97,109,115,123,132]
	HM (meningeal metastasis)			1	[98]
	D3H2LN (pleural effusion)	Luciferase		5	[54,77,111,112,133]
		Luciferase	miR	1	[90]
	B02 (pleural effusion)		miR	1	[58]
	BrM (brain metastasis)	Luciferase	miR	1	[121]
	1833/TGL (metastatic bone)			1	[106]
	IBC3	GFP	miR KD	1	[81]
	4175 LM2	Luciferase		3	[34,101,102]
	K8ikd		miR	1	[116]
MCF-7	DCIS	Luciferase		1	[98]
	Parental			4	[39,45,70,111]
		GFP		1	[70]
		GFP	miR	1	[55]
		Luciferase		2	[74,114]
		Luciferase	miR	3	[57,89,134]
			Pri-miR	1	[43]
			miR sponge	2	[43,48]
			miR, miR regulators, Anti-miR	7	[52,56,75,88,120,124,131]
MCF-10CA1h	Parental	GFP-luciferase	miR	1	[85]
SKBR3	Parental	Luciferase	Anti/miR sponge	1	[118]
	TR (trastuzumab resistant)		miR sponge	1	[42]
SUM-	149	GFP	miR KD	1	[81]
	159pt			1	[99]

**Table 3:** Murine models used in miRNA experiments.

Model	No. of Experiments	References
Metastatic	61	
– Tail vein intravenous – Intrarterial	42	[22,23,25,29,30,31,32,42,43,48,49,50,52,53,54,55,56,58,64,65,66,67,70,78,80,81,83,85,86,87,89,90,93,94,95,104,109,110,116,119,127,129]
– Left ventricle	6	[51,98,114,121,131,133]
– Intratibial	4	[51,71,130,131]
– Intrapulmonary	1	[73]
– Spontaneous after orthotopic	5	[57,79,102,128,135]
– Spontaneous after orthotopic with primary mass removed	3	[23,34,112]
Orthotopic	40	[22,24,25,26,27,29,32,35,40,44,47,50,54,59,63,65,66,76,77,78,79,82,84,85,86,88,95,96,98,99,100,101,105,113,116,117,123,125,132,134]
Xenograft (subcutaneous)	33	[28,30,31,33,36,39,41,42,43,45,46,49,53,56,68,69,70,72,73,75,91,92,93,97,103,107,111,115,118,122,124,126,136]

**Table 4:** miRNA delivery system in mice.

Vehicle	Formulation	miRNA	No. of Experiments	References
NP (*n* = 29)	Lipid (LNP)	miR-34a, -124, -143, 186-3p, -203, -214-3p and -379-5p	8	[36,37,59,60,66,123,133,135]
	Gold (Au)	miR-155, -708 and -96/-182	3	[27,34,35]
	Silico (SiO2)	miR-34a	1	[99]
	Magnetic (MN)	miR-10b, -376B and 21/-145/-9	5	[26,39,111,112,122]
	Polymers	miR-21, -34a, -145 and -21/10b	8	[28,33,38,41,65,68,108,125]
	RNA	miR-21, -205/-221	4	[47,82,113,132]
miRNA chemically modified (*n* = 12)	Mimic	miR-489 (CMM489), miR-34a (FolamiR)	2	[109,115]
	AgomiR AntagomiR	miR-16-1-3p, -100 and -338-3p	3	[24,29,32]
	Small-molecule inhibitors	miR-10b (“Linifanib”), -21(“AC1MMYR2”), -210 (“TargapremiR”) and -544	6	[40,63,94,96,97,105]
	Peptide nucleic acid (PNA)	miR-155	1	[45]
EV (*n* = 6)	Exosome	miR-21, -159, -210, -335, -4443 and let-7	6	[46,61,69,74,101,107]-
Plasmid (*n* = 62)	Lentiviral	miR-1, 23a, -27b, -29a, -33a, -33b, -96, -100, -101, -124, -125a, -125b, -133a-3p, -133b, -138, -150, -190, -206, -211-5p, -218-5p, -373, -429, -442a, -452, -454-3p, -455-3p, -494, -509, -543, -548j, -630, -940, -1204, -200 family and -30 family	44	[28,30,32,33,35,37,39,40,41,42,43,44,45,46,47,48,49,51,52,53,56,57,58,59,60,61,62,63,64,65,66,67,68,69,70,71,72,73,74,75,87,88,100]
	DNA	miR-1, -29/-30, 106b-5p, -135/203, -196a, -205, 361-5p, -497, -590-3p, -567, let-7a-5p, -14q32-encoded miRNAs, -191/425 and -200 family	16	[25,30,31,43,44,52,56,76,83,91,103,110,114,117,119,126]
	Circular inhibitor	miR-21/-223	1	[118]
	Inducible plasmid	miR-301a-3p	1	[75]
Other (*n* = 5)	Antiviral miRNA	mja miR-34, -35	2	[64,93]
	Circular RNA	miR-1233-3p, -3942	2	[124,131]
	Pterostilbene	miR-105	1	[92]

NPs: nanoparticles; EVs: extracellular vesicles.

**Table 5:** Therapy effects in mice models following miRNA delivery.

Therapy Effects	Vehicles	miRNAs Studied	No. of Experiments	References
Tumor growth (*n* = 35)	NP	miR-203, 143, -145, -186-3p, -379, -376B 5p, -34a, -21 and -205/-221	16	[28,33,36,37,38,41,47,59,60,82,99,113,122,123,132,138]
	EV	miR-335, -159, -21 and let-7	4	[46,61,101,107]
	miRNA chemically modified	Linifanib (miR-10b), “Small mol.1” (miR-544), FolaramiR-34a and TargapremiR-210	4	[96,97,105,115]
	Plasmid	miR-455-3p, -100, -442a, -125a-5p, -138, -27b, 196a, -567, cirBulg21/223 and -301a-3p	10	[44,72,75,77,84,88,100,117,118,134]
	Other	Shrimp miR-34	1	[93]
Tumor growth and lung metastasis (*n* = 39)	Plasmid	miR-101, -1, -211-5p, -96, -494, -1204, -133b, -206, -30a-5p, -548j, --141, -190, -125b, -33a, -33b, -29/30, -361-5p, let-7a, -191/-425 and -200 family	24	[22,23,25,30,31,42,43,50,53,54,56,70,73,78,79,85,86,91,95,98,116,120,127,128]
	NP	-708, -96/-182, -34a, 10b; -124 and -21/10b	7	[26,34,35,65,66,108,135]
	miRNA chemically modified	CMM489 (miR-489), miR-338-3p, AntagomiR-100, AntagomiR-16-1-3p and AC1MMYR2 (miR-21 inhibitor)	6	[24,29,32,40,63,109]
	Other	Pterostilbene, circular RNA	2	[92,124]
Lung metastasis (*n* = 20)	Plasmid	miR-630, -150, -133b, -133a-3p, -10b, -452, -543, -29a, -373, -23a, -454-3p, -590-3p, -106b-5p, -200 family members and 14q32-encoded miRNAs	16	[48,49,52,55,57,62,80,83,87,89,90,102,104,110,119,129]
	NP	miR-10b	1	[112]
	Other	miR-35; -1233	2	[64,67]
	miRNA chemically modified	miR-21	1	[94]
Bone metastasis (*n* = 9)	Plasmid	miRNA-124, -125b, -135/203; 429, -940, -205, -218-5p and -30 family members	8	[51,58,71,76,106,114,130,131]
	NP	miR-214-3p	1	[133]
Liver metastasis (*n* = 1)	EV	miR-4443	1	[74]
Brain metastasis (*n* = 2)	Plasmid	miR-141, -509	2	[81,121]
Biodistribution (*n* = 6)		miR-200c, -34a, -155, -10b	6	[27,39,45,103,111,125]
Angiogenesis (*n* = 3)		miR-497, -210, -125-5p	2	[69,73,126]

NP: nanoparticle; EV: extracellular vesicle.

**Table 6:** Number of experiments for each specific modality and aim.

Imaging	Aim	No. of Experiments	References
Bioluminescence	Metastasis engraftment and growth	44	[23,30,31,32,42,43,48,49,50,52,53,54,55,56,58,59,62,64,67,70,76,78,79,80,81,83,87,90,91,93,94,102,104,110,112,114,116,119,121,127,128,129,131,133]
	Tumor engraftment and growth	26	[22,26,44,50,69,74,76,77,82,84,88,92,96,97,100,101,103,105,107,111,117,118,123,130,132,134]
	Tumor growth and metastasis	14	[24,25,34,40,57,63,73,85,86,95,102,106,108,120]
	Vector uptake and intracellular target repression	2	[74,115]
	VEGFR2 transcription in transgenic mice	1	[126]
Fluorescence	Vector biodistribution	19	[26,28,34,36,38,46,60,61,65,66,68,69,107,111,112,113,122,132,135]
	Tumor growth	3	[35,72,124]
	Vector persistence after intratumoral injection	2	[41,99]
	Cell tracking	1	[83]
	Molecular beacon for specific miR detection	1	[125]
µCT	Evaluation of osteolytic lesions	5	[51,71,106,130,133]
	Pulmonary metastasis	2	[22,35]
MRI	Nanoparticle biodistribution	3	[28,39,107]
	Adjacent tissue invasion from primary mass	1	[74]
PET/CT – [18F]-FDG	Tumor growth and metabolism	2	[36,37]
	Pulmonary metastasis	1	[51]
Radiography	Osseous metastasis analysis	2	[58,131]
HFUS	Tumor growth	1	[41]
	Therapy delivery microbubble-mediated	1	[33]
PA	Specific identification of miR	1	[27]
SPECT – [^99m^Tc]-labeled probe	Specific identification of miR	1	[45]

FDG: fluorodeoxyglucose; HFUS: high-frequency ultrasonography; µCT: microcomputed tomography; MRI: magnetic resonance imaging; PA: photoacoustic; PET/CT: positron emission tomography/computed tomography; SPECT: single-photon emission computed tomography; and VEGFR2: vascular endothelial growth factor receptor 2.
